# Supplementary figures and images for: Nuclear position modulates long-range chromatin interactions
Source: PLoS Genet. 2022 Oct 7;18(10):e1010451. doi: 10.1371/journal.pgen.1010451 (PMC9581366; doi:10.1371/journal.pgen.1010451)

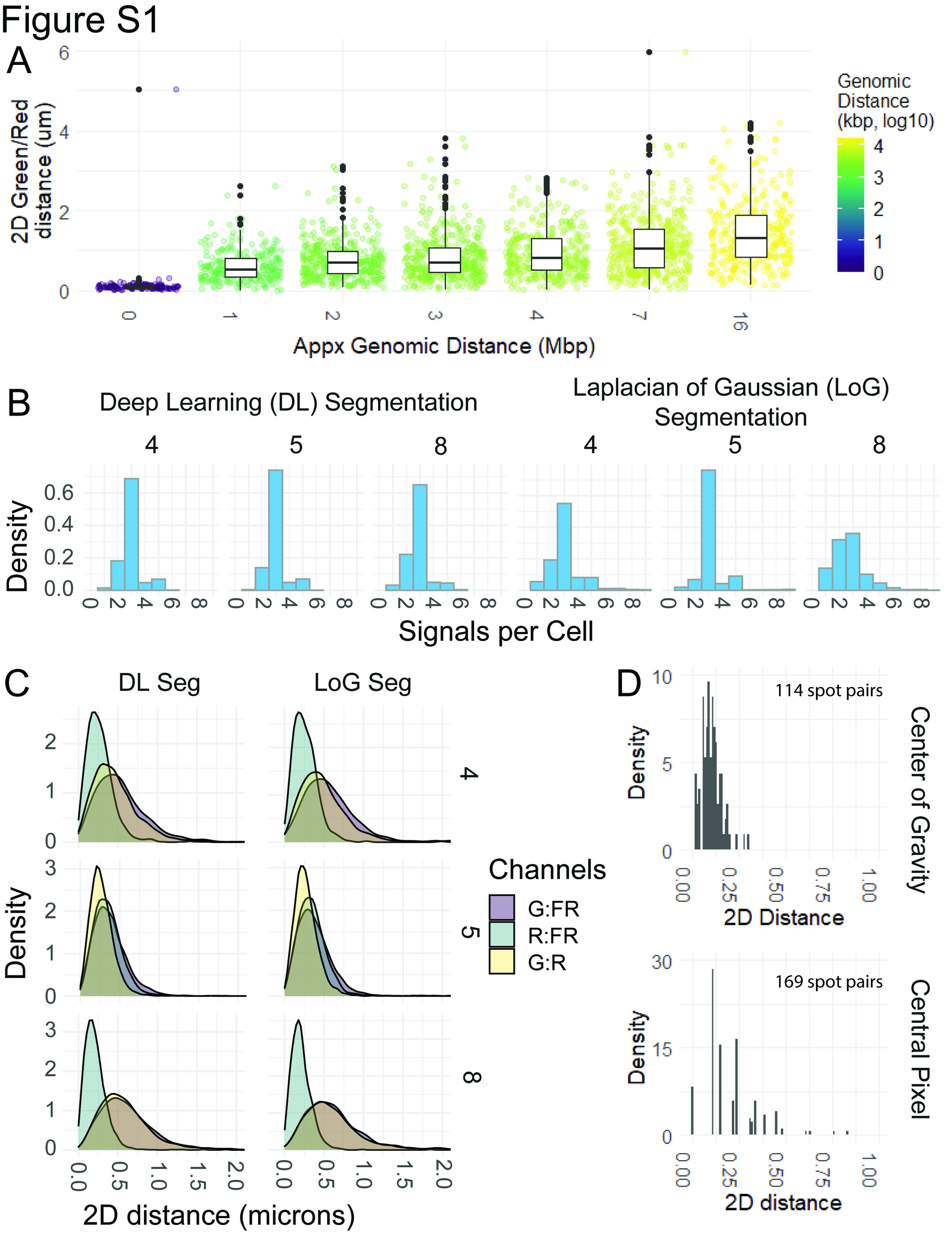

Supplement: S1 Fig — A: Box and jitter plots showing distribution of distances for a selection of probe pairs including a costained control locus (Distance 0) as well as several probe pairs at genomic distances up to ~16 Mbp. Center-to-center genomic distance is color-coded. B: Comparative spots per cell for spots segmented from three representative wells with a deep-learning based published model [47] as compared to and traditional Laplacian of Gaussian-based segmentation. Wells selected for a breadth of FISH quality, from very high signal to noise (well 5) to borderline (well 8). C: Spot-to-spot distance distributions calculated from the spots segmented in (B). Color-coded by pair of channels within the well. D: Spot-to-spot distances for the 120:120 costained locus with spot positions assigned by center of gravity (top) or central pixel (bottom). (TIF) [file pgen.1010451.s001.tif]

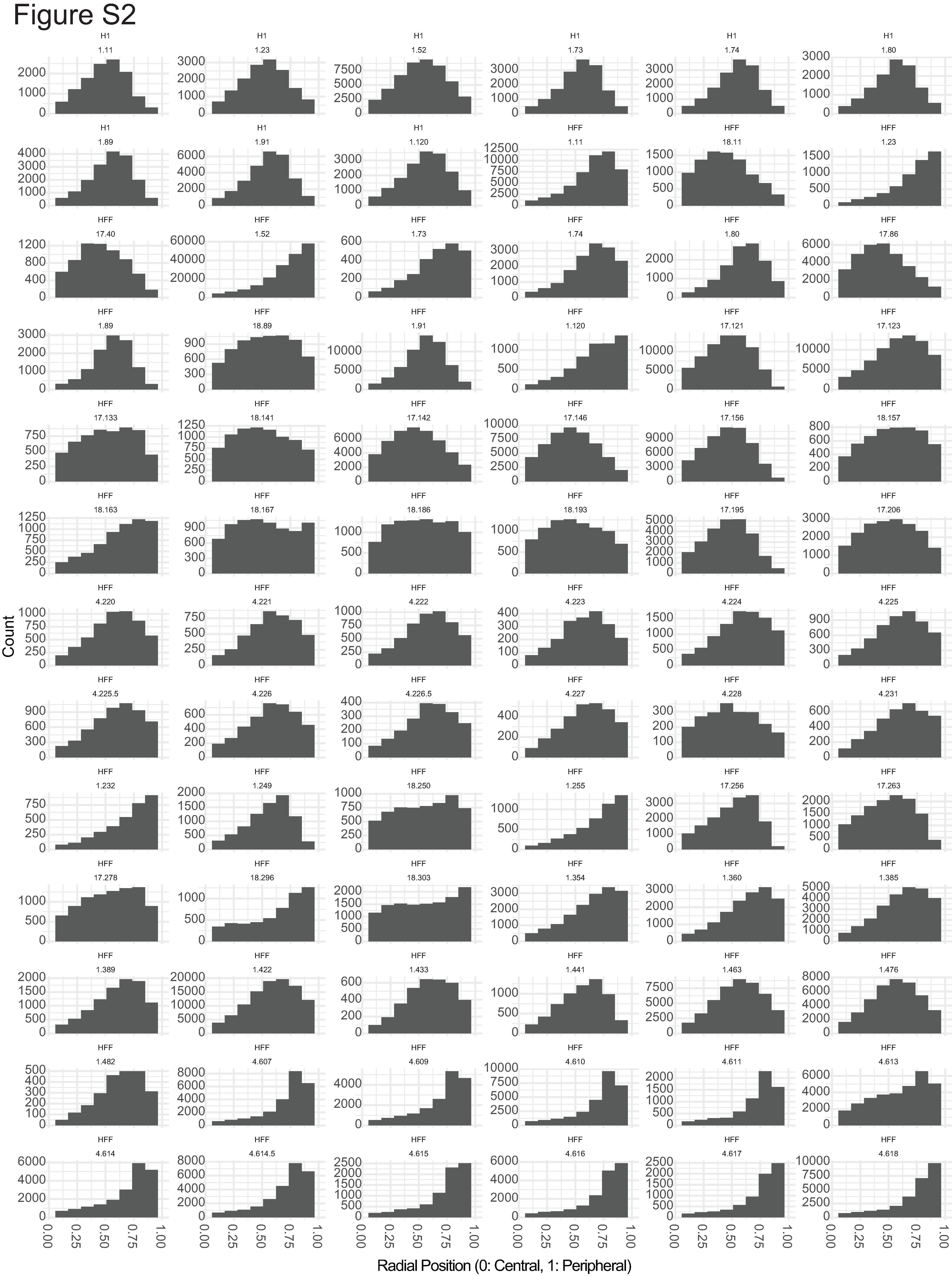

Supplement: S2 Fig — Histograms showing radial position of all spots at all loci in both cell types. Legend for each panel is: cell type (top line), chromosome and probe number (bottom line; chr.probe). For radial position, a value of 0 is fully central and a value of 1 is fully peripheral. (TIF) [file pgen.1010451.s002.tif]

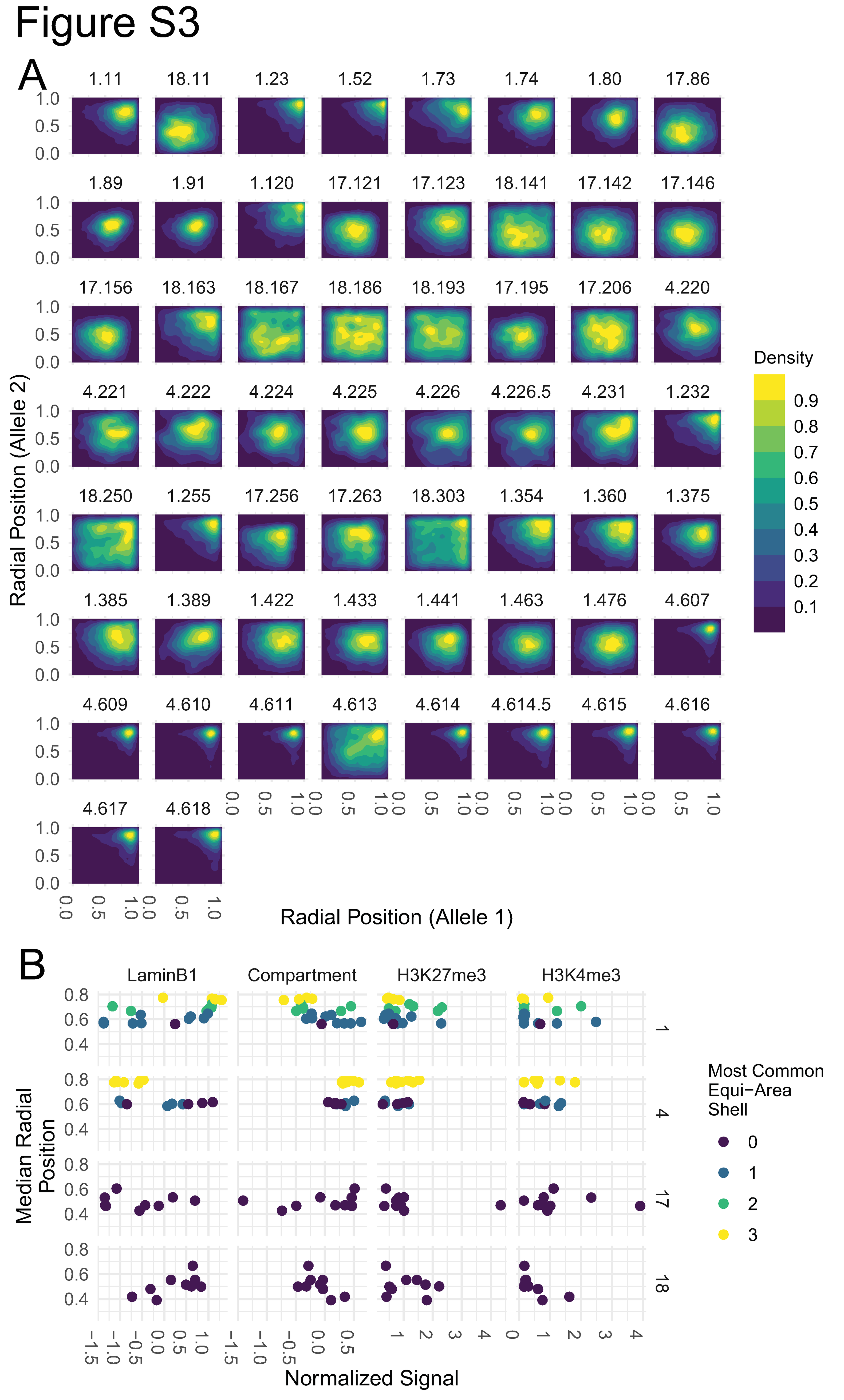

Supplement: S3 Fig — A: Normalized 2D Density plots showing radial position at one homolog (arbitrarily selected) on the x-axis and radial position at the other homolog on the y-axis. Probe and chromosome as marked. B: Scatterplots showing median continuous normalized radial position vs. sequencing metrics: LaminB1 enrichment by Dam-ID, Chromatin Compartment in Micro-C, H3K27me3 and H3K4me3 signal in ChIP-seq. Color-coded by most common radial shell. (TIF) [file pgen.1010451.s003.tif]

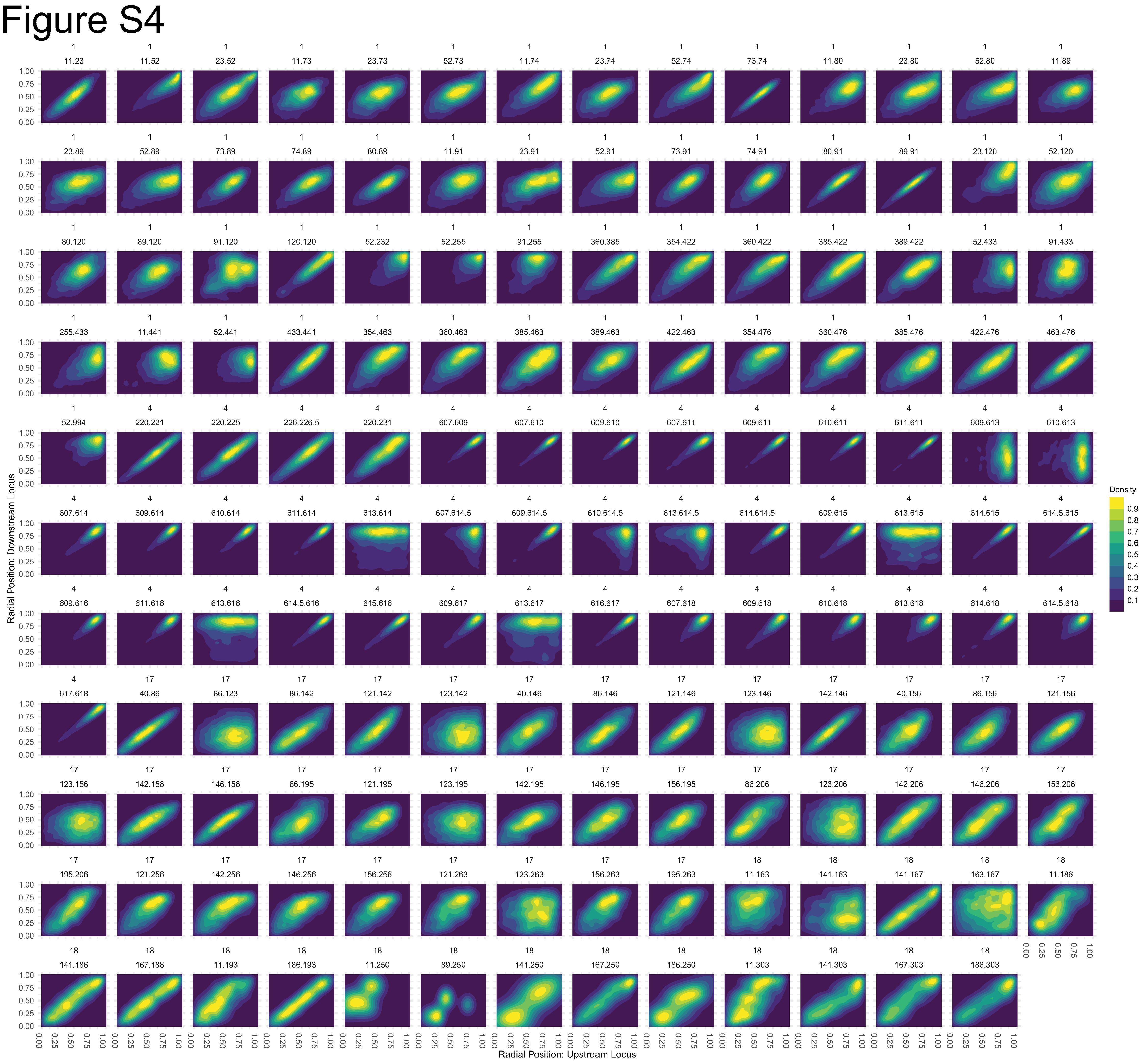

Supplement: S4 Fig — 2D Density plots showing radial position at one locus in a pair on the x-axis and the other locus on the y-axis. Probe pair and chromosome as marked. (TIF) [file pgen.1010451.s004.tif]

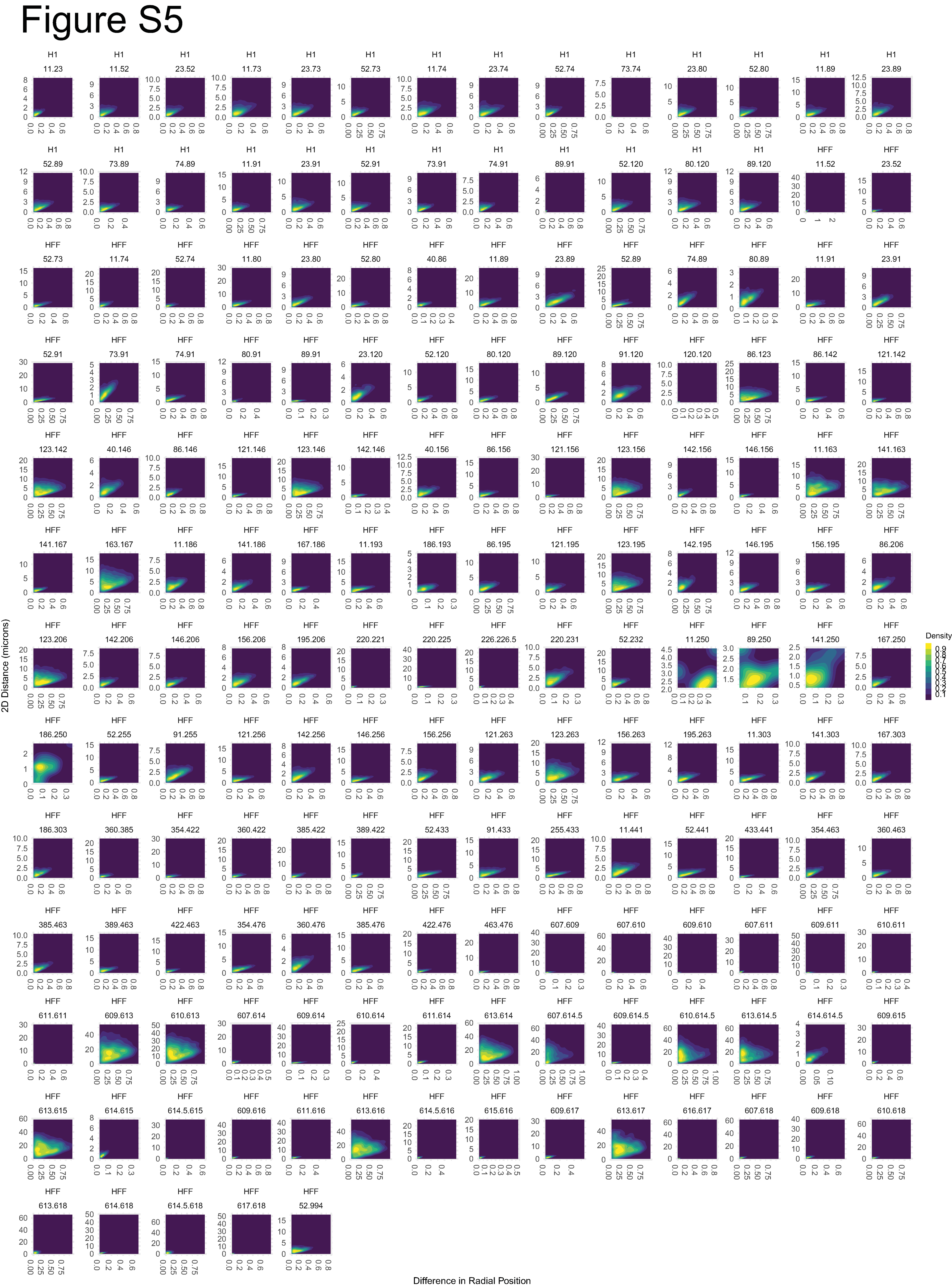

Supplement: S5 Fig — 2D Density plots showing difference in radial position between loci in a pair on the x-axis and spatial distance between loci the y-axis. Probe pair and chromosome as marked. (TIF) [file pgen.1010451.s005.tif]

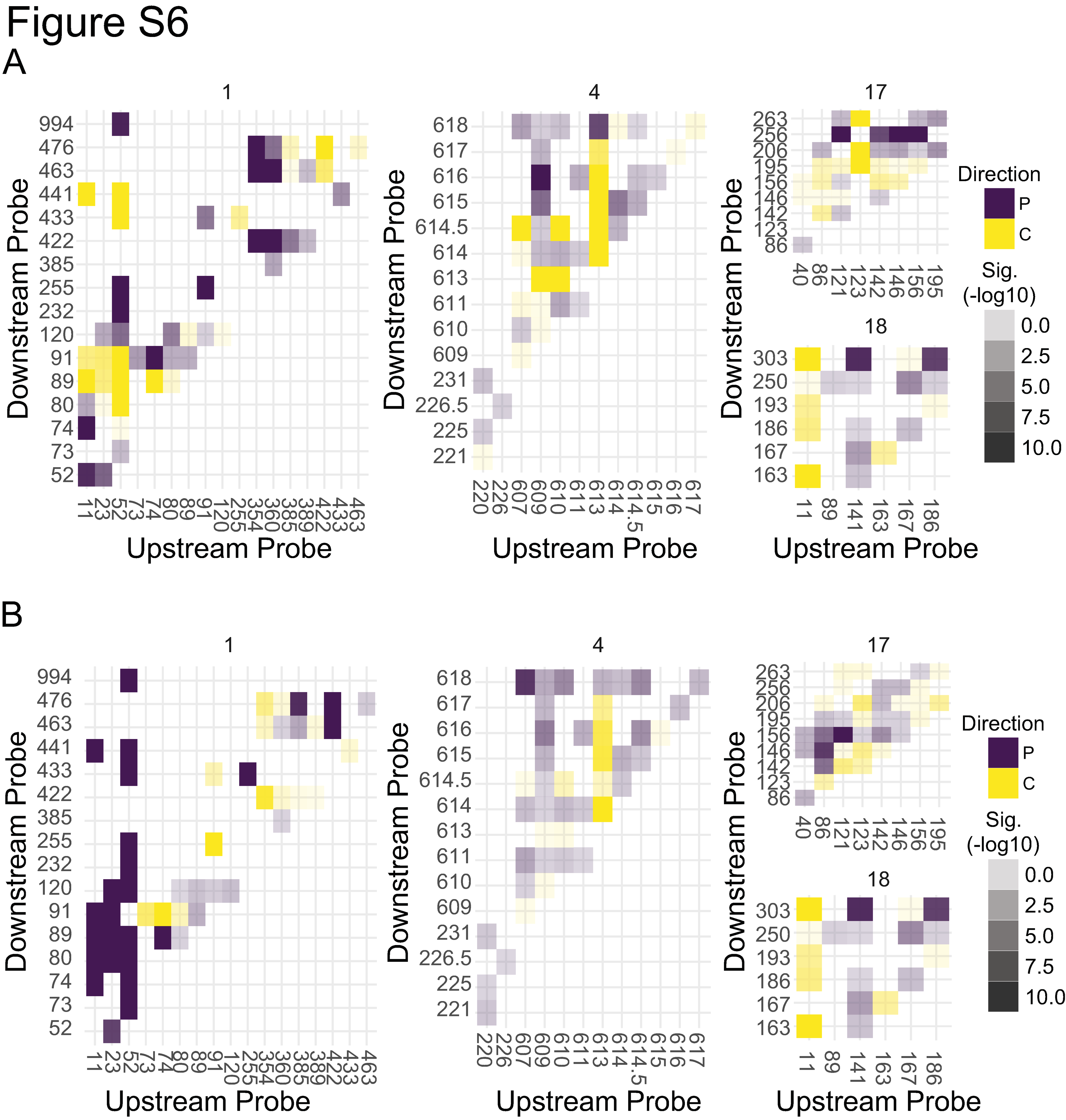

Supplement: S6 Fig — Heatmaps for each pairwise interaction on a chromosome, with probe number (approximate genomic position) on both x and y axis. Intensity of color (alpha) is significance (as -log10(p-value) in the ANOVA test). Color is direction (as slope of line of best fit). (TIF) [file pgen.1010451.s006.tif]

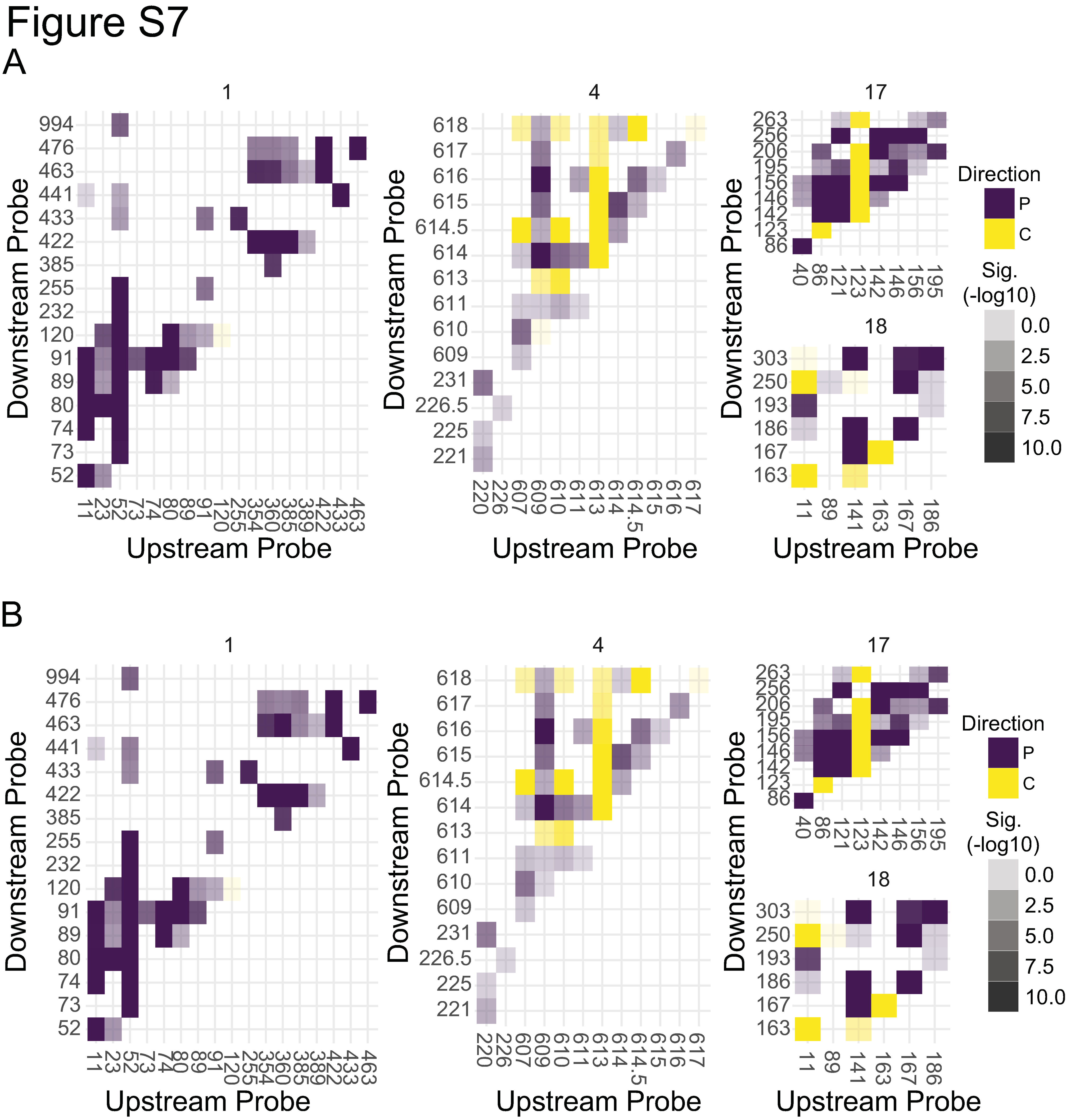

Supplement: S7 Fig — Heatmaps for each pairwise interaction on a chromosome, considering only those instances where both loci in the pair are found within the same radial shell. Probe number (approximate genomic position) is on both x and y axis. Intensity of color (alpha) is significance (as -log10(p-value) in the ANOVA test). Color is direction (as slope of line of best fit). (TIF) [file pgen.1010451.s007.tif]

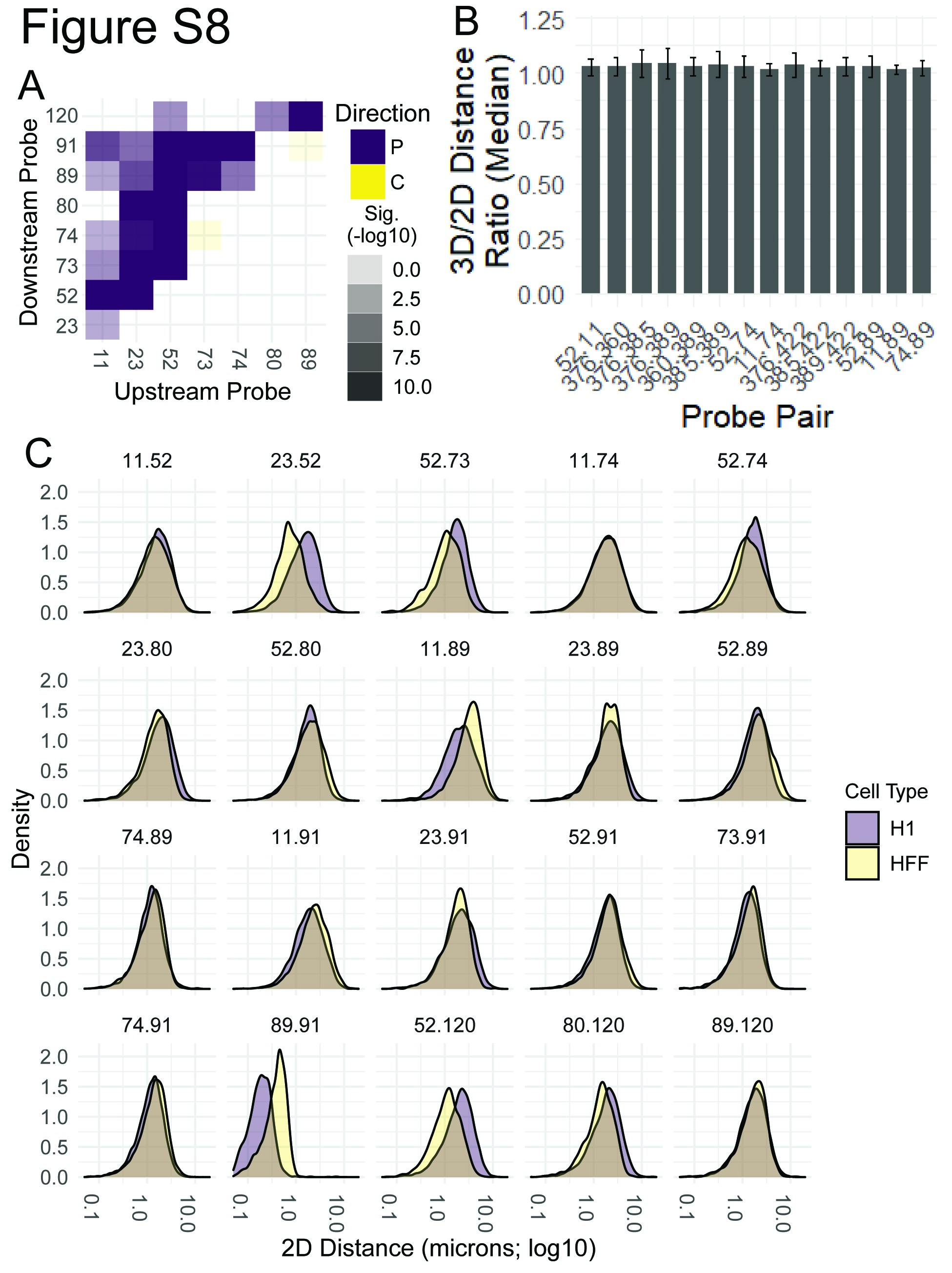

Supplement: S8 Fig — A: Heatmap showing association between radial position of downstream spot and spatial distance between spots, for each pairwise interaction on a chromosome, with probe number (approximate genomic position) on both x and y axis. Intensity of color (alpha) is significance (as -log10(p-value) in the ANOVA test). Color is direction (as slope of line of best fit). B: Median ratio between 2D and 3D distances for 14 pairs of loci on chromosome 1 in columnar stem cells. Error bars are median absolute deviation in 3D/2D ratio. C: Probability density functions showing 2D spatial distance distribution for all pairs examined in both HFFs and H1s. Pair is as marked, color-coding by cell type. (TIF) [file pgen.1010451.s008.tif]
